# Green design of a paper test card for urinary iodine analysis

**DOI:** 10.1371/journal.pone.0179716

**Published:** 2017-06-28

**Authors:** Nicholas M. Myers, Ivan C. Leung, Sean W. McGee, Kathleen Eggleson, Marya Lieberman

**Affiliations:** 1 Department of Chemistry and Biochemistry, University of Notre Dame, Notre Dame Indiana, United States of America; 2 Department of Electrical Engineering, University of Notre Dame, Notre Dame Indiana, United States of America; Institut de recherche pour le developpement, FRANCE

## Abstract

When young children do not receive adequate amounts of the micronutrient iodine in their diet, their growth and cognitive development can be impaired. Nearly every country in the world has programs in place to track iodine intake and provide supplemental iodine if needed, usually in the form of fortified salt. The iodine nutrition status of a population can be tracked by monitoring iodine levels in urine samples to see if the median value falls in the range of 100–300 micrograms of iodine per liter of urine (μg I/L), which indicates adequate or more than adequate iodine nutrition. Many low and middle-income countries (LMIC) do not have a laboratory capable of carrying out this challenging assay, so samples must be sent out for assay in external labs, which is expensive and time-consuming. In most LMIC, population iodine surveys are carried out every 5–10 years, which limits the utility of the data for program monitoring and evaluation. To solve this problem, we developed a field-friendly paper test card that uses the Sandell-Kolthoff reaction to measure urinary iodine levels. A blind internal validation study showed that 93% of samples (n = 60) of iodide in an artificial urine matrix were categorized correctly by visual analysis as deficient, adequate, or excessive for levels set forth by the World Health Organization. Quantitative measurements based on computer image analysis had an error of 40 ± 20 μg I/L (n = 35 for samples in the calibration range) and these results categorized 88% of the samples (n = 60) correctly. We employed lifecycle analysis principles to address the known toxicity of arsenic, which is an obligatory reagent in the Sandell-Kolthoff reaction. Disposal of the cards in a landfill (their most likely destination after use) could let arsenic leach into groundwater; toxicity characteristic leaching procedure (TCLP) tests showed that the level of arsenic leached from the cards was 28.78 ppm, which is above the United States Environmental Protection Agency’s limit of 5 parts per million for solid waste. We integrated a remediation module into the card. This module contains oxone, to oxidize As(III) to As(V) oxyacids, and the iron oxide goethite. TCLP testing showed that the leachable amount of arsenic was reduced by at least 97.6%—from 28.8 ppm to lower than 0.7 ± 0.7 ppm (n = 20). This upstream intervention rendered the test card suitable for landfilling while retaining its functionality to perform a critical public health evaluation.

## Introduction

Millions of children are at risk for cognitive impairment that can be prevented with iodized table salt at a cost of $0.05 per person per year [1–4]. However, it is necessary to make sure the salt is consumed; this is done by measuring the iodine levels in urine samples collected from a representative subset of a population [5]. The technical difficulty of analyzing trace amounts of iodine in urine makes it difficult for low and middle-income countries (LMIC) to conduct surveys frequently and hinders the monitoring and evaluation of iodine supplementation programs. The World Health Organization deems a population as iodine-deficient if the median urinary iodine value obtained during a survey is less than 100 micrograms of iodine per liter of urine (μg I/L), adequate if it is 100–199 μg I/L, above requirements if it is 200–299 ug I/L, and excessive if greater than or equal to 300 μg I/L [6]. Only about 100 labs worldwide are registered with the Centers for Disease Control and Prevention’s Ensuring the Quality of Urinary Iodine Procedures (EQUIP) program, which provides standardized urinary iodine samples to check that a lab’s in-house method is producing accurate results [7–9]. Ideally, population surveys would be continuous, but instead they are done about every 5–10 years within many countries [10].

A promising approach to increase global capacity for monitoring iodine supplementation programs is to develop a field-screening tool that can measure urinary iodine, or at least categorize the iodine in urine samples according to the WHO’s classification system. The WHO’s interpretation of “adequate” and “above requirements” for iodine health status are similar, and are consistent with a multi-center study published by Zimmermann showing no adverse effects from the "above requirements" range[11], so we grouped these ranges together to span 100–299 ug I/L for the purposes of developing this technology. This would allow the interpretation of the test card’s output caused by the urinary iodine to be read as deficient, adequate, or excessive in levels of iodine. A fast turn-around time would also enable iodization programs to evaluate the effectiveness of nutritional interventions in real time. We decided to develop a paper analytical device (PAD) as a screening tool to categorize urinary iodine levels. PADs are inexpensive, simple for non-experts to use, and require no electricity [12]. Thus, they hold great promise where public health need co-occurs with a paucity of resources and infrastructure for laboratory-based techniques.

We designed a paper test card to accurately measure physiologically relevant iodine levels in simulated urine. The card gives a colorimetric readout that a person can interpret by eye to obtain categorization of urine samples, or can image with a cell phone camera for quantification by computer image analysis. The colorimetric reaction relies on the Sandell-Kolthoff (SK) reaction, a kinetically slow reaction between Ce^4+^ and As^3+^ that is catalyzed by iodide (Scheme 1. Reactions 1 and 2) [13]. The fading of the yellow Ce^4+^ color is difficult to monitor by eye, particularly in urine samples that are strongly colored, but ferroin can be used as a redox indicator to enable visual readout (Scheme 1. Reactions 3 and 4). Previous laboratory methods make use of this indicator to allow visual analysis or digital readout by a plate-reader [14–16]. Only one field-friendly technique for urinary iodine assay is reported in the literature, but the specificity obtained during field validation was 61% [17].

1. ${4\text{ Ce}}^{4+}{+\;4\text{ I}}^{-}\to {2\text{ I}}_{2}{\;+\;4\text{ Ce}}^{3+}$
2. ${5\text{ H}}_{2}{\text{O }+\;2\text{ I}}_{2}{+\text{ As}}_{2}{\text{O}}_{3}\to {10\text{ H}}^{+}{+4\text{ I}}^{-}{+\;2\text{ AsO}}_{4}^{3-}$
3. ${\text{Ferroin}}^{2+}{\;(\text{red})\;+\text{ Ce}}^{4+}\to {\text{Ferroin}}^{3+}{\;(\text{blue})\;+\text{ Ce}}^{3+}$
4. ${4\text{ Ferroin}}^{3+}{\;(\text{blue})\;+\text{ As}}_{2}{\text{O}}_{3}{\;+\;5\text{H}}_{2}\text{O}\to {4\text{ Ferroin}}^{2+}{\;(\text{red})\;+\;2\text{AsO}}_{4}^{3-}{\;+\;10\text{ H}}^{+}$

Scheme 1. Sandell-Kolthoff reaction [13].

1. Ce^4+^ oxidizes the iodide in urine to iodine.
2. Iodine is reduced back to iodide by arsenite. The iodide-catalyzed reaction between Ce^4+^ and As^3+^ is known as the Sandell-Kolthoff reaction [13].
3. The reaction is tracked visually using ferroin, [Fe(*o*-phen)_3_]^2+^. The solution is blue while excess Ce^4+^ is present.
4. Excess arsenite regenerates ferroin. The solution turns red to signal the completion of the SK reaction.

Since the Sandell-Kolthoff method requires arsenic, disposing of its waste is problematic in the low resource settings where the card would likely be employed. The most likely fate of the cards would be a landfill with no barriers or monitoring. To responsibly develop this technology, we set a goal to reduce the amount of leachable arsenic below levels set by regulatory standards. The Environmental Protection Agency’s toxicity characteristic leaching procedure (TCLP) for solid wastes defines an acceptable limit to be less than 5 parts per million arsenic in a volume of leachate determined by a function of the test card’s weight [18]. Even though this is a regulatory requirement for the United States, it is a useful guideline in the development of this technology for application in LMIC, where environmental regulations may not address arsenic waste. The goal of this upstream intervention in the product design is to reduce the harm at the end of the product lifecycle without compromising its performance [19].

## Results and discussion

### Test card design

**Fig 1:**
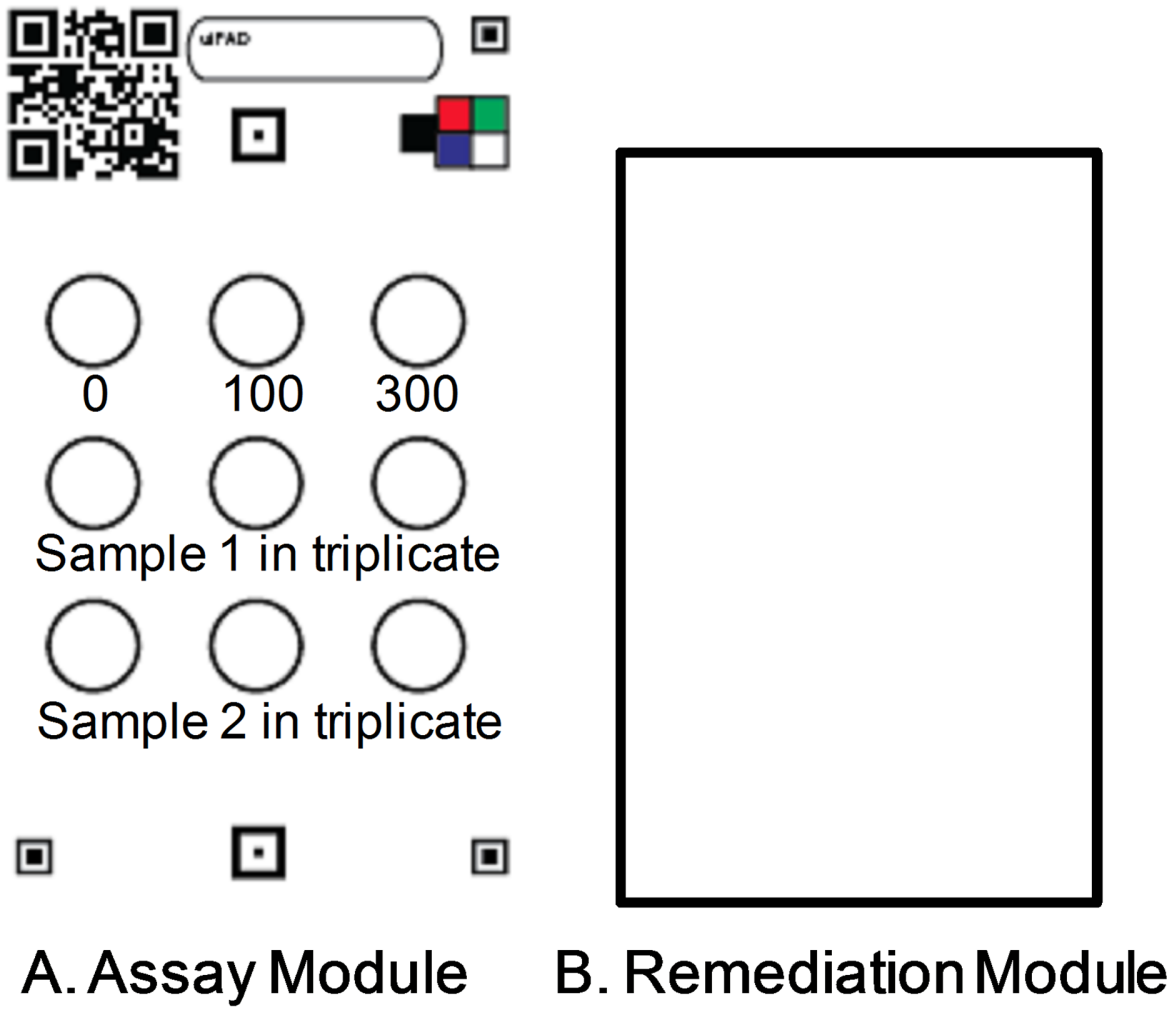
Layout of the test card. A. Assay Module. The 0, 100, and 300 μg I/L internal iodide standards in the top row are used to determine the concentrations of the samples in the bottom rows. B. Remediation Module. The rectangular area is loaded with reagents to bind arsenic so it cannot leach into groundwater. After the assay is complete, the remediation module is folded on top of the assay module to render the test card non-toxic. Fiducial marks and color standards are printed on the card to facilitate automated image analysis.

The test card is divided into an assay module and a remediation module (Fig 1). The assay module has nine analysis areas which are defined by wax [20]; three are used for standards and six are used for samples. At the time of analysis, the user prepares the test card by pipetting onto each circle solutions that are provided in a test kit. The exact recipe is in the methods section. There is a 25 fold reduction in the amount of arsenic needed to perform the analysis when compared to the gold standard UV-vis method because smaller volumes of solutions are needed (S1 Table) [21]. The defined reaction areas confine the assay solutions and produce superior reproducibility in color production when compared to borderless reaction areas [22]. When the user pipets the last solution, which contains arsenite, the SK reaction is initiated. The final reagent can be loaded into all 9 circles in about 30 seconds using a single channel automatic pipette, or a multi-channel pipet can be used to initiate the nine reactions in about 10 seconds. After 3 minutes, the 0, 100, and 300 μg I/L standards attain distinctive colors, and the results from the triplicate measurement of the sample can be interpreted visually or the card can be photographed to record the data for later analysis. The final step is to render the test card nonhazardous with the aid of the remediation module. The user does this by adding a capsule of goethite to the remediation module, which is preloaded with an oxidant. The remediation module is then pressed face-to-face with the assay module, and they are folded together. At this point they can be placed into the trash. The cost of the materials for the assay and remediation modules is about $0.20USD each. See S2 and S3 Tables for a cost analysis.

### Development of the assay module

**Fig 2:**
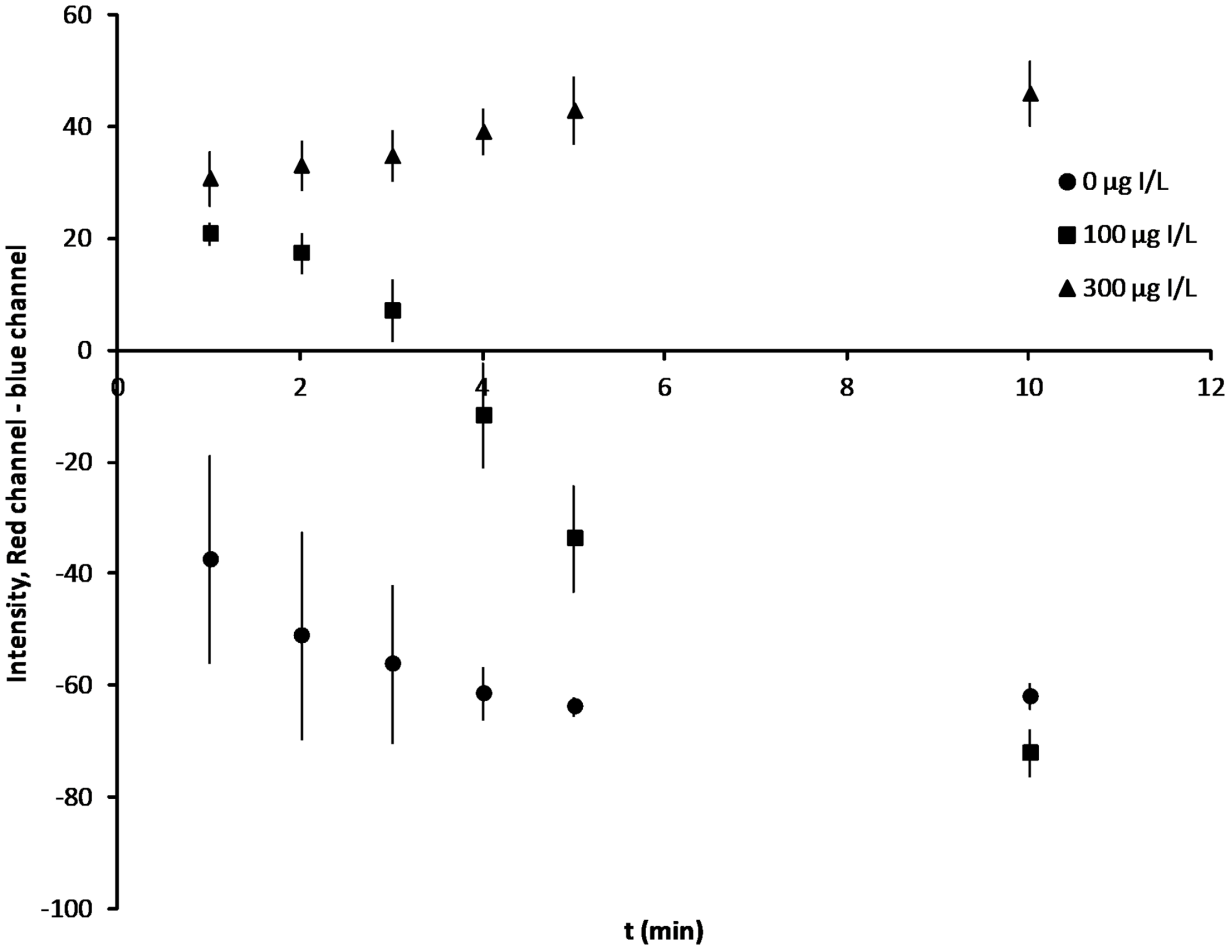
Progression of the Sandell-Kolthoff reaction on paper. The test card’s internal standards change color over time and are distinguishable by ImageJ analysis of cell phone pictures taken 1–10 minutes after the start of the reaction. The best distinction is achieved 3–5 minutes into the reaction. Error bars show the SD of 3 test cards.

The formulation of the device was optimized to produce a readable output in a short period of time. Several colored species (Ce^4+^, ferriin, and ferroin) are present during the course of the reaction, so we evaluated different metrics for measuring the reaction progress. For image analysis, single channel measurements (S1 and S2 Figs) were not as effective as the difference between the red and blue channels (Fig 2). This metric gives the best distinction amongst solutions that contain the most important iodine levels of 0, 100, and 300 μg I/L at a reaction time of 3–5 minutes. The 100 μg I/L test solution exhibited an unexpected trend in color development, becoming bluer over time instead of redder. Ceric arsenate precipitates during the preparation of the test card because the solution is not sufficiently acidic (i.e., a basic arsenic solution is pipetted into an acidic cerium solution) [5]. The ceric arsenate dissolves over time. If the ceric ion dissolves faster than the SK reaction can consume it, the solution stays blue. For the solution containing 300 μg I/L standard, the ceric ion is consumed quickly, and the excess As(III) reduces ferriin to ferroin, giving the solution a red color.

### Validation of the assay module

**Fig 3:**
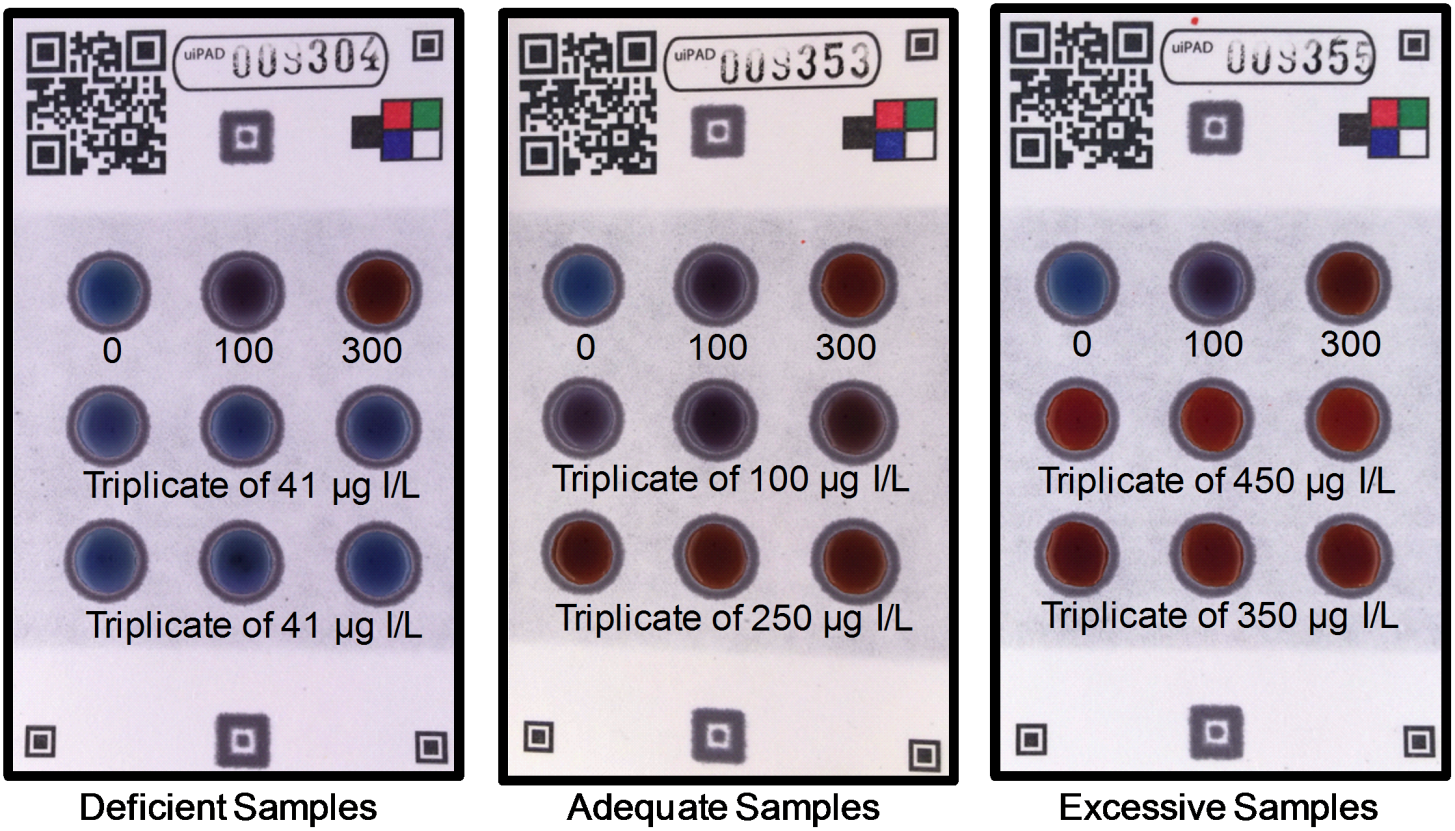
The test card response to various levels of iodide at 3 minutes. The blank standard appears blue while the 100 μg I/L standard looks purple, and the 300 μg I/L standard is red. Each unknown was applied to three circles in a row; the concentration of iodide in the sample solution is shown below the row. The samples are visually categorized to contain < 100, 100–299, or ≥ 300 μg I/L.

The analytical metrics of the assay module were determined by a blind internal study using 30 different solutions of artificial urine containing physiologically relevant concentrations of iodide, urea, chloride, sodium, and potassium [23]. The preparer coded the solutions and gave them to two different researchers to analyze on the test cards. The researchers ran the solutions independently and took pictures of the test cards in a light box using an iPhone 5s (S7 Fig). They read the device by visually comparing the color of the samples to that of the standards after 3 minutes of reaction time (Fig 3). Pooling all reads together, 56 out of the 60 test card reads (93%) were correctly categorized as deficient, adequate, or excessive in iodine content (Table 1). All 20 deficient samples were identified correctly. Two of the mis-categorizations were borderline errors (i.e., a 300 μg I/L sample was categorized as adequate rather than excessive, and a 110 μg I/L sample was categorized as deficient rather than adequate). This data gives a weighted Cohen’s kappa value, K, of 0.926, meaning there is good categorization of the data even when chance agreement is accounted for [24]. The accuracy of visual categorization means that the cards could be used in field settings where electrical power and internet connectivity are not reliable.

**Table 1:** Visual and computer image analysis of the test cards.

	Visual analysis, μg I/L			Computer image analysis, μg I/L		
True values, μg I/L	< 100	100–299	≥ 300	< 100	100–299	≥ 300
< 100	20	0	0	16	4	0
100–299	1	17	2	0	20	0
≥ 300	0	1	19	0	3	17
Cohen’s kappa, K	0.926			0.825		

For the visual analysis, 56 out of 60 samples (93%) were properly categorized, and all iodine deficient samples were recognized. For the computer image analysis, 53 out of 60 samples (88%) were properly categorized. The weighted Cohen’s kappa values show both analysis methods categorize samples correctly.

**Fig 4:**
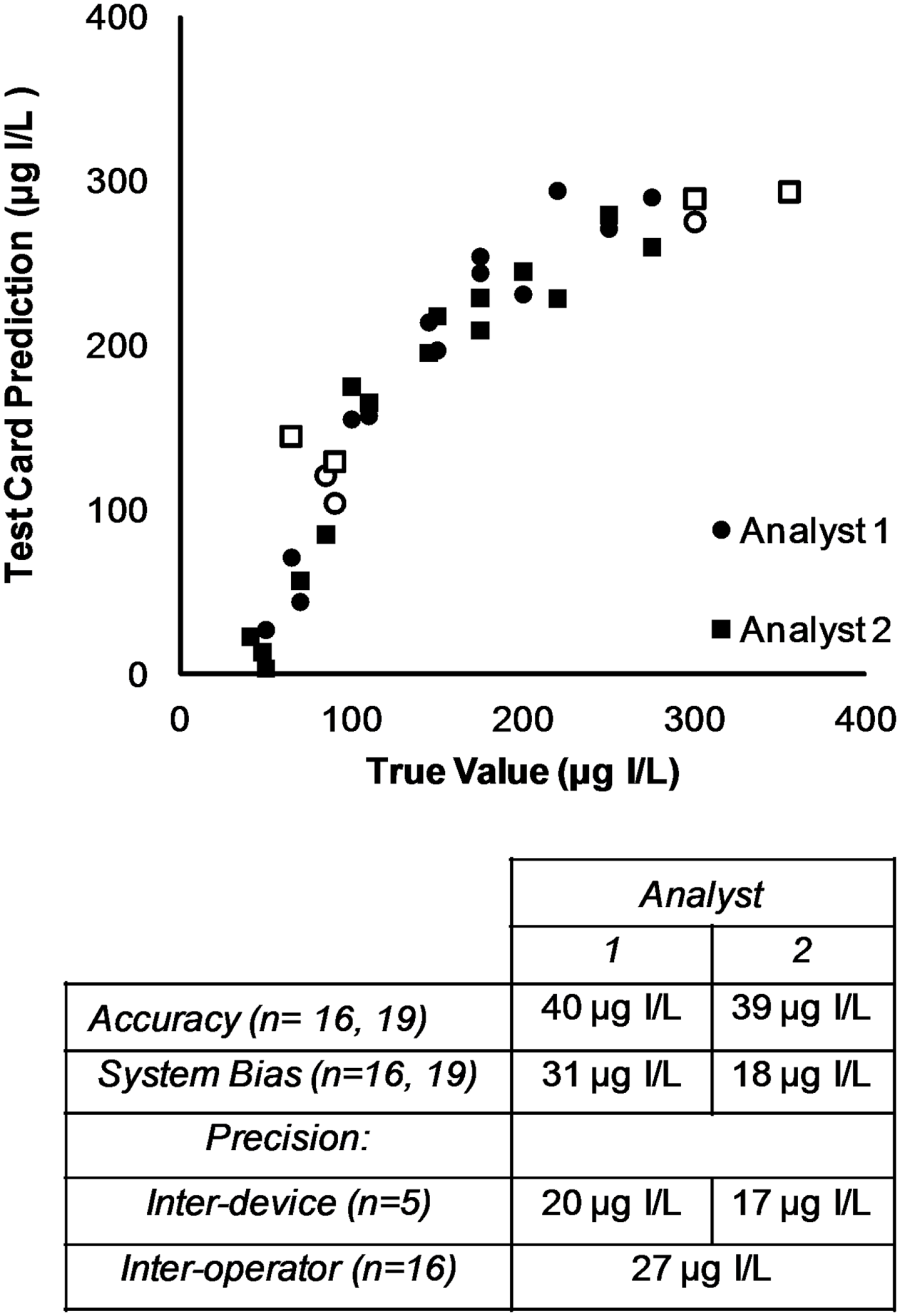
Accuracy plot for ImageJ analysis of the test card. Mis-categorized results are shown as empty circles or squares, correctly categorized results as filled circles or squares. All metrics are expressed as μg I/L.

Computer image analysis has several potential advantages over visual analysis, including greater objectivity, less dependence on the visual acuity of the operator, and greater ability to archive and share raw data and test results. Pictures of the cards were acquired in a home-built light box and analyzed in ImageJ. The internal standards on each test card were used to create a calibration curve against which the samples were compared. A quantitative readout was generated and each sample was categorized as deficient, adequate, or excessive. This yielded 53 out of 60 correct predictions (88%). See Table 1. The weighted K value was 0.825, which again shows good categorization for the technique. Nearly all mis-classifications happened when the true value was near a cutoff boundary (Fig 4). The visual categorization out-performed the computerized categorization, but both yielded good results. This suggests that there are additional color parameters that could be taken into account by the computerized analysis to bring its performance level up to that of the human eye.

The quantitative readouts produced by ImageJ analysis can be assessed directly instead of forcing them into a qualitative response. The data was further assessed to understand the accuracy of individual test card measurements. Only 35 card responses fell into the calibration range of 0 to 300 μg I/L and were used in the following metrics. 17 excessive results were disregarded as well as 8 results with negative values (the negative values were within the error of the y-intercept). The accuracy (average of absolute errors), system bias (average of errors), inter-device precision (standard deviation of a 150 μg I/L standard, n = 5), and inter-operator precision (the average difference in results obtained by two analysts when using the same solution, n = 16) were calculated (Fig 4). Both analysts performed the same on accuracy (40 μg I/L) and inter-device precision (20 μg I/L), and both over-estimated the iodine content of the test solution (on average, Analyst 1 was 31 μg I/L high and Analyst 2 was 18 μg I/L high). Inter-operator precision was 27 μg I/L on average when they each analyzed the same solution (S4 Fig). The test cards tend to under-estimate solutions containing less than 100 μg I/L, but over-estimate solutions containing 100–300 μg I/L (S5 Fig). The sensitivity of the assay is highest in the 50–300 μg I/L range, which spans both threshold values for the desirable amount. Above and below these concentrations there is little variation in the color of the indicator at t = 3–5 min, so quantification becomes unreliable. Capturing more images over a longer period of time could increase the dynamic range. Despite the strength of the test card’s analytical metrics, an individual’s iodine nutrition status should not be assessed via a spot urine sample.

Development of the automated image analysis for the test cards also facilitates data storage and sharing with nutritional monitoring programs. Analyzing cell phone pictures of paper devices is possible in low resource settings [25–27], and cell phones are ubiquitous in Africa [28]. Pictures can be taken within a light box to impart consistent lighting for analytical measurements. (S7 Fig.)

Population status is assessed using the median value of UIC obtained during a survey. If the results from each individual card are reasonably accurate, the categorization for the median card will characterize the urinary iodine status for the population analyzed. The true median value for the data set was 175 μg I/L. For the median card, analyst 1 obtained 242.5 μg I/L, and analyst 2 obtained 223.1 μg I/L (S3 Fig). Both the numerical values and the categorization of the median card correctly predicted the population status as "not iodine deficient."

The next step will be development of a more field-friendly urine pre-treatment procedure. Urine is a complex matrix with more than 60 chemical species exceeding 10 ppm [23]; some of these species are known to interfere with the SK reaction [29]. When the SK reaction is performed in a lab setting the urine is boiled with strong acids and oxidizers to remove interferences [21], but we are trying to avoid this procedure for field use. Filtering the urine through activated carbon has been reported to remove interferents [30]. If filtration does not work, matrix effects may be compensated for by using the method of standard additions, the feasibility of which has already been demonstrated on a paper substrate [31].

### Development of the arsenic remediation module

The amount of arsenic needed for the paper implementation of the SK assay was reduced by 25-fold when compared to the gold standard UV-vis method (S1 Table); however, the amount on the card still poses a health risk because it can leach into the environment after disposal. A toxicity characterization leaching procedure (TCLP) carried out on the assay module of the card showed levels of 28.78 ppm As in the leachate, which categorizes the assay module as hazardous waste according to EPA regulations. The arsenic can be put into a non-leachable form after the test is complete by binding it to iron oxide [32]. Arsenic and iron oxides have a high affinity for each other, but the binding works best when the pH is slightly acidic, the arsenic is fully oxidized, and the iron oxide is in a specific mineral phase called goethite [33]. The pH condition was already met by the assay solutions, so we added an oxidizing agent to the remediation module to convert leftover arsenite to arsenate and to combat reducing conditions sometimes found in landfills. To accomplish this, 60 mg of potassium peroxymonosulphate (Oxone^®^) is stored in the paper. After the assay module is used, goethite is sprinkled over the paper, and it is folded over to bring all the remediation reagents into contact. The TCLP experiment can then be repeated to determine the leachable arsenic level after on-card remediation. Iron oxides can be stored in paper fibers (S6 Fig), which allows for a user-friendly system of folding over one module onto the other, but more development work is needed to fit the goethite in the test card area. The cost of the materials to make the remediation module is about $0.20USD (S3 Table).

### Performance of the arsenic remediation module

TCLP measurements were made according to EPA SW-846 Test Method 1311 with arsenic analysis by ICP-OES. The theoretical starting arsenic level in the leachate was 28.82 ppm, and when cards were subjected to the TCLP without any remediation efforts, 28.78 ppm arsenic was measured.

**Fig 5:**
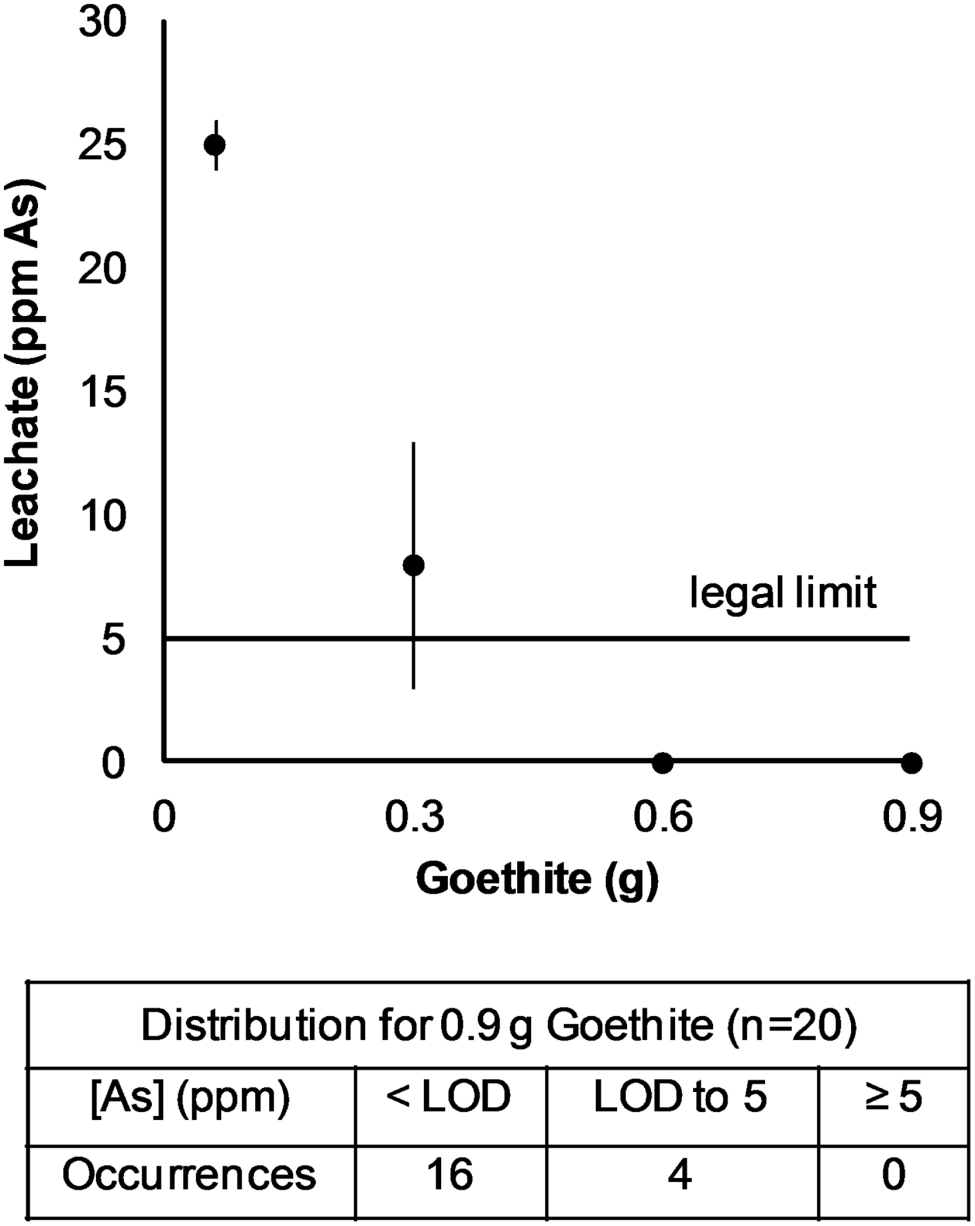
Toxicity characteristic leaching procedure results for test cards with various amounts of goethite. At least 0.6 g goethite must be used to mitigate the leachate to acceptable arsenic levels. For the 0.9 g level, n = 20 and for all others n = 3. LOD = 0.028 ppm As. All error bars are shown, but some are too small to see on this scale.

Of 20 test cards subjected to the remediation procedure (Fig 5), 16 had undetectable levels of arsenic in the leachate, so the remediation level was estimated using the method LOD of 0.028 ppm; more than 99.9% of the arsenic was removed from these samples. (See “Arsenic remediation” in the Methods section). The average arsenic leached from the remaining four cards was 0.7 ± 0.7 ppm. This corresponds to 97.6% of the arsenic being absorbed. All 20 samples met the regulatory requirement of producing leachate with arsenic levels below 5 ppm, so the assay module plus the remediation module would be classified as nonhazardous waste by EPA standards.

## Conclusion

We designed an easy-to-use paper test card to quickly measure physiologically relevant iodine concentrations without the need for lab equipment, specialized instruments, or reliable electrical power. Over the 0–500 μg I/L range in an artificial urine matrix, the test cards correctly classified 88% of samples by computerized image analysis and 93% by visual analysis. Computer image analysis can extract quantitative data from the test card; the accuracy was 40 μg I/L, and the precision was 20 μg I/L.

The arsenic levels required for the test card classified it as toxic waste by US regulatory standards, so we addressed the risk associated with the analysis by incorporating a remediation module into the card design. The arsenic level in leachate from the test card was reduced to such a low concentration by the remediation module that it could not be detected by ICP-OES in most samples, and all 20 cards tested were well below the EPA regulatory limits. Without compromising the efficacy of the Sandell-Kolthoff reaction, the end product was rendered less hazardous to the person handling its disposal as well as the entire community over time by preventing arsenic from leaching into the environment. These risks could have been ignored by citing public health imperatives or by acknowledging the lack of resources for proper disposal in LMIC. Conversely, concerns about the toxicity of the chemical wastes could have stymied further development of this potentially useful field test. The cost to develop the remediation module was well worth the benefit of eliminating a possible barrier to implementation in developing world settings. The risk associated with this dual-module paper test card has been reduced as much as is reasonably possible, and further development is ethically justified by its potential use for the identification of a serious public health threat.

## Materials and methods

### Chemicals

Urea (J.T. Baker); sodium chloride (Macron); potassium chloride (Fisher); potassium iodide (Amresco); arsenic trioxide (Alfa Aesar); ceric ammonium nitrate (Alfa Aesar); 1,10-phenanthroline (Amresco); iron sulfate (J.T. Baker); sulfuric acid, trace metal grade (Fisher); sodium hydroxide (BDH); 1000 ± 2 ppm iodide standard, ICP grade (Fluka lot BCBP1989V); goethite, characterized by XRD (Aldrich); nitric acid (BDH); glacial acetic acid (Fisher); oxone (Alfa Aesar); test solutions were diluted with deionized water; 18 M-Ohm water was used for ICP-OES work.

### Precautions to prevent iodide contamination

All glassware, reagents, and paper were handled with care to prevent iodide contamination. This included wearing disposable gloves and working in a hood lined with freshly laid out absorbent towels. Glassware was washed with 5% v/v nitric acid.

### Simulated urine recipe

Water was spiked with 15,000 ppm urea, 3800 ppm chloride, 1800 ppm sodium, and 1200 ppm potassium to mimic urine. These values are within physiological ranges [23]. This diluent was used to create standards for the validation study.

### Fabrication of test card

The test card is designed using Adobe Illustrator with art boards that are 8.5” x 11”, which accommodates printing onto Ahlstrom 319 paper with commercially available printers. One layer of the art board is used to create the fiducial marks, QR code, color standard, lettering, and serialization zone; all are printed with a laser printer. Another layer of the art board is used to create the reaction zone circles, remediation zone, and backside barrier; all are printed with a wax printer (Xerox ColorQube 8570N). Specific dimensions can be found in the accompanying Adobe Illustrator file. The cards are baked at 100°C for 14 minutes and a subset are tested with water to ensure the barriers are sealed; if the barriers leak, the cards are baked for 3 additional minutes.

### Running and analyzing the test card

Solutions are pipetted onto the test card in the following order: 1) In the top row, add to individual circles 50.0μL of 0, 100, and 300 μg/L iodide standards (in a synthetic urine matrix), 2) In the second row, add 50.0 μL of sample to each circle, 3) In the third row, add 50.0 μL of sample to each circle, 4) Add to every circle 2.0 μL of 0.12 M ferroin, 5) Add to every circle 4.0 μL of 0.4 M ceric ammonium nitrate in a 0.5 M sulfuric acid solution, and 6) Add to every circle 10.0 μL of 0.2 M As_2_O_3_ in a 0.15 M NaOH solution. Leaving the card on a flat surface, move it back and forth about 1 cm at a rate of 2 Hz to facilitate mixing. The shaking requires some practice because if it is too vigorous the solution will spill out of the reaction zones, and the user must start over in this case. The reaction area can hold about 75 μL of solution before it escapes from the shaking motion. For this study, after 3 minutes of shaking, a picture of the card was taken in a light box with an iPhone 5s. The light box was equipped with 2 plug-in strips of white LED lights that each had an output of 162 lumens and rated 82 on the color-rendering index. The images were analyzed visually by comparing the samples to the internal standards and picking the appropriate category. The images were also analyzed in ImageJ. The images were split into red, green, and blue channels. The average un-weighted gray value over the entire reaction area was measured. The blue channel was subtracted from the red channel, then for each test card a linear calibration curve was generated from the response of the standards; the concentration of the samples were determined against the calibration curve.

### Blind internal validation

A researcher volumetrically diluted ICP-grade iodide standard to 30 different concentrations using synthetic urine as the diluent. The samples were coded and given to two analysts who ran them on the test cards independently on different days. The analysts interpreted the test cards by eye and with ImageJ [34], and then submitted the results to the original researcher who knew the true concentrations.

### Arsenic remediation

The remediation module was pre-loaded and dried with 60 mg Oxone^®^, then loaded with 0.9 g goethite at the time of analysis. It was flipped on top of the assay module, folded together, and placed into a glass jar to perform the Environmental Protection Agency’s toxicity characterization leaching procedure (TCLP).[18] The leachate had to be diluted by a factor of ten and the instrument lines washed with 10% v/v nitric acid solution for 3 minutes in between injections to avoid salt and rust buildup in the ICP-OES. The test solution was filtered and analyzed by a Perkin Elmer Optima 8000 ICP-OES. The plasma was viewed down the axial axis, and the analytical wavelength monitored was As 193.696 nm. The torch was placed at the -4 position. The plasma gas flow rate was 15 L/min and the auxiliary gas flow was 0.2 L/min. The nebulizer was set to 0.7 L/min and power at 1400 W. The peristaltic pump flowed at 2.0 mL/min. The negative controls had no detectable levels of arsenic (n = 3). The LOD for As containing solutions was estimated to be 0.0028 ppm (3*standard deviation of replicate injections of a 0.1 ppm As standard, n = 5); a ten-fold dilution was performed on the leachate, so the method LOD was 0.028 ppm. The recovery of the 28.82 ppm starting arsenic level in the leachate was 99.9% (n = 3). Quality control samples were performed every 5^th^ injection, and they always analyzed within 3% error.

## Supporting information

S1 Table Arsenic waste by method. 7.5/0.3 = 25. It takes 25 times more arsenic by mass to analyze one sample by the UV-vis method than by the test card.
(DOCX)

S2 Table Cost analysis of assay module. (DOCX)

S3 Table Cost analysis of remediation module. (DOCX)

S1 Fig ImageJ analysis of standards run on the test card, red channel. Only the red channel intensity was measured. The error bars are 1 SD of 3 replicate test zones. There is not good distinction at any time.
(DOCX)

S2 Fig ImageJ analysis of standards run on the test card, blue channel. Only the blue channel intensity was measured. The error bars are 1 SD of 3 replicate test zones. There is not good distinction at any time.
(DOCX)

S3 Fig Population health status prediction. The median values lie within the dotted box, and all are within the 100–299 μg I/L range. Both analysts predict the same population health status as the true value.
(DOCX)

S4 Fig Inter-operator precision. Each solution was analyzed by 2 people on separate test cards. There is no trend for one analyst to consistently predict higher concentrations than the other. There are 15 points above and 15 points below the x-axis.
(DOCX)

S5 Fig Residual plot for the computerized image analysis of the test card. The residual plot shows a systematic underestimation of iodide solutions that truly contain lower levels while solutions which contain 100–300 μg I/L are overestimated. The readings above 300 μg I/L are extrapolated.
(DOCX)

S6 Fig Iron oxides storage in paper fibers. Scanning electron microscopy images. All images are to the same scale. A. An individual fiber of paper can be seen. B. When iron oxides are loaded onto the test card, they form clusters around the paper fibers. C. After adding the arsenic containing test solution, the iron oxides remain around the paper fibers.
(DOCX)

S7 Fig Light box construction. (DOCX)
