# Supplementary material for: Green design of a paper test card for urinary iodine analysis
Source: PLoS One. 2017 Jun 28;12(6):e0179716. doi: 10.1371/journal.pone.0179716 (PMC5489186; doi:10.1371/journal.pone.0179716)
Supplement: S1 Table — 7.5/0.3 = 25. It takes 25 times more arsenic by mass to analyze one sample by the UV-vis method than by the test card. (DOCX) [file pone.0179716.s001.docx]

**S1 Table. Arsenic waste by method.**

| **Method** | **Volume (L)*** | **Concentration (M)** | **Amount (mg)** |
| --- | --- | --- | --- |
| UV-vis | 2 x 10^-3^ | 0.025 M As_2_O_3_ | 7.5 |
| Test Card | 10 x 10^-6^ | 0.2 M As_2_O_3_ | 0.3 |

7.5/0.3 = 25. It takes 25 times more arsenic by mass to analyze one sample by the UV-vis method than by the test card.

*Volume of arsenic containing test solution.
