# Supplementary material for: Green design of a paper test card for urinary iodine analysis
Source: PLoS One. 2017 Jun 28;12(6):e0179716. doi: 10.1371/journal.pone.0179716 (PMC5489186; doi:10.1371/journal.pone.0179716)
Supplement: S2 Table — (DOCX) [file pone.0179716.s002.docx]

**S2 Table. Cost analysis of assay module.**

| **Expenditure** | **Cost per test card ($USD)** |
| --- | --- |
| Ahlstrom 319 Paper | 0.05 |
| Wax | 0.03 |
| Packaging Materials | 0.05* |
| Consumables | 0.05* |
| Chemicals | 0.02* |
| Total | 0.20 |

*Assumes 20 cards/pack.
