# Supplementary material for: Green design of a paper test card for urinary iodine analysis
Source: PLoS One. 2017 Jun 28;12(6):e0179716. doi: 10.1371/journal.pone.0179716 (PMC5489186; doi:10.1371/journal.pone.0179716)
Supplement: S1 Fig — Only the red channel intensity was measured. The error bars are 1 SD of 3 replicate test zones. There is not good distinction at any time. (DOCX) [file pone.0179716.s004.docx]

**S1 Fig. ImageJ analysis of standards run on the test card, red channel.** Only the red channel intensity was measured. The error bars are 1 SD of 3 replicate test zones. There is not good distinction at any time.
