# Supplementary material for: Green design of a paper test card for urinary iodine analysis
Source: PLoS One. 2017 Jun 28;12(6):e0179716. doi: 10.1371/journal.pone.0179716 (PMC5489186; doi:10.1371/journal.pone.0179716)
Supplement: S3 Fig — The median values lie within the dotted box, and all are within the 100–299 μg I/L range. Both analysts predict the same population health status as the true value. (DOCX) [file pone.0179716.s006.docx]

**S3 Fig. Population health status prediction.** The median values lie within the dotted box, and all are within the 100-299 μg I/L range. Both analysts predict the same population health status as the true value.
