# Supplementary material for: Green design of a paper test card for urinary iodine analysis
Source: PLoS One. 2017 Jun 28;12(6):e0179716. doi: 10.1371/journal.pone.0179716 (PMC5489186; doi:10.1371/journal.pone.0179716)
Supplement: S4 Fig — Each solution was analyzed by 2 people on separate test cards. There is no trend for one analyst to consistently predict higher concentrations than the other. There are 15 points above and 15 points below the x-axis. (DOCX) [file pone.0179716.s007.docx]

**S4 Fig. Inter-operator precision.** Each solution was analyzed by 2 people on separate test cards. There is no trend for one analyst to consistently predict higher concentrations than the other. There are 15 points above and 15 points below the x-axis.
