# Supplementary material for: Green design of a paper test card for urinary iodine analysis
Source: PLoS One. 2017 Jun 28;12(6):e0179716. doi: 10.1371/journal.pone.0179716 (PMC5489186; doi:10.1371/journal.pone.0179716)
Supplement: S5 Fig — The residual plot shows a systematic underestimation of iodide solutions that truly contain lower levels while solutions which contain 100–300 μg I/L are overestimated. The readings above 300 μg I/L are extrapolated. (DOCX) [file pone.0179716.s008.docx]

**S5 Fig.** **Residual plot for the computerized image analysis of the test card.** The residual plot shows a systematic underestimation of iodide solutions that truly contain lower levels while solutions which contain 100-300 μg I/L are overestimated. The readings above 300 μg I/L are extrapolated.
