# Supplementary material for: Green design of a paper test card for urinary iodine analysis
Source: PLoS One. 2017 Jun 28;12(6):e0179716. doi: 10.1371/journal.pone.0179716 (PMC5489186; doi:10.1371/journal.pone.0179716)
Supplement: S6 Fig — Scanning electron microscopy images. All images are to the same scale. A. An individual fiber of paper can be seen. B. When iron oxides are loaded onto the test card, they form clusters around the paper fibers. C. After adding the arsenic containing test solution, the iron oxides remain around the paper fibers. (DOCX) [file pone.0179716.s009.docx]

| 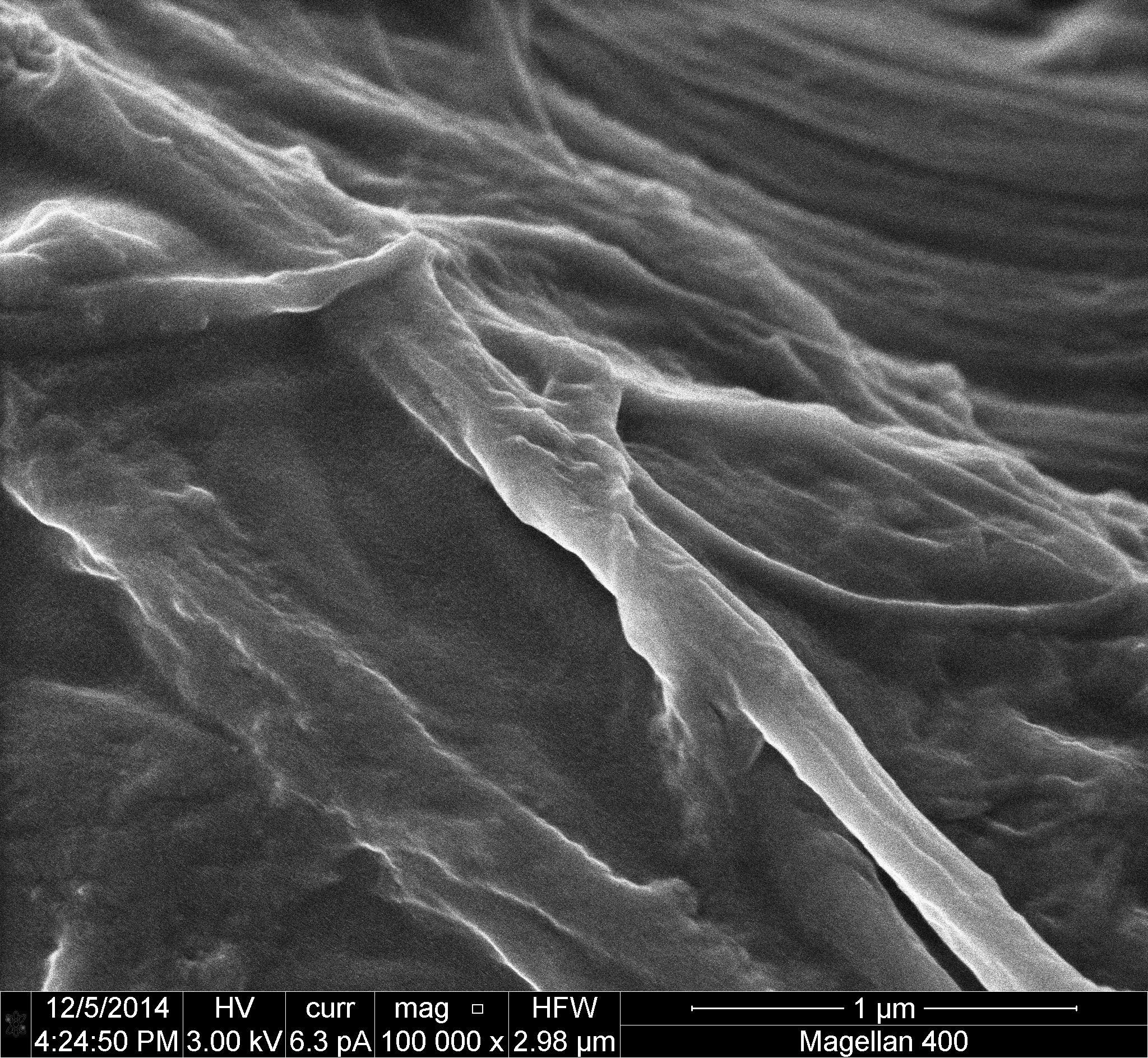  1 μm | 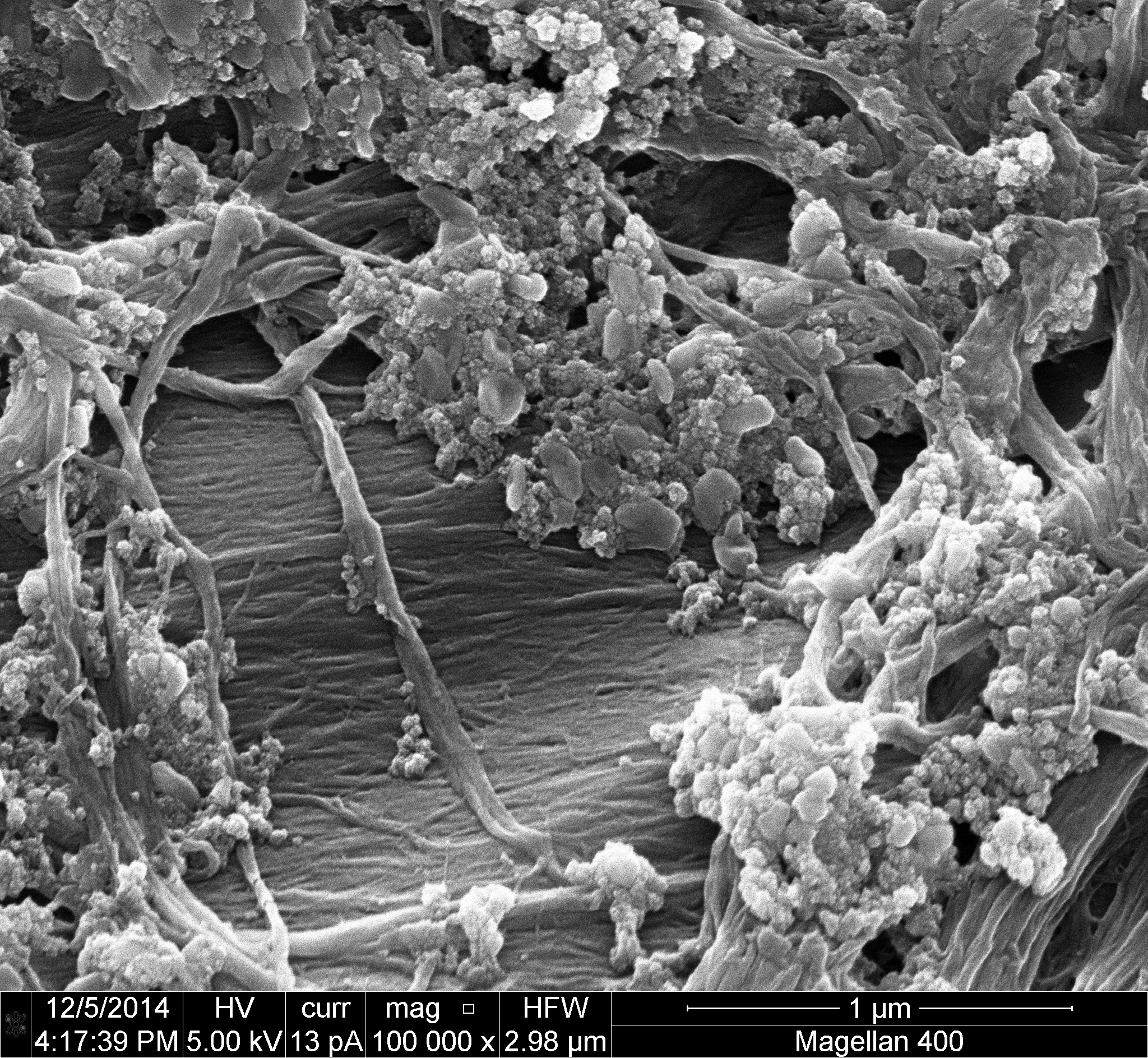  1 μm | 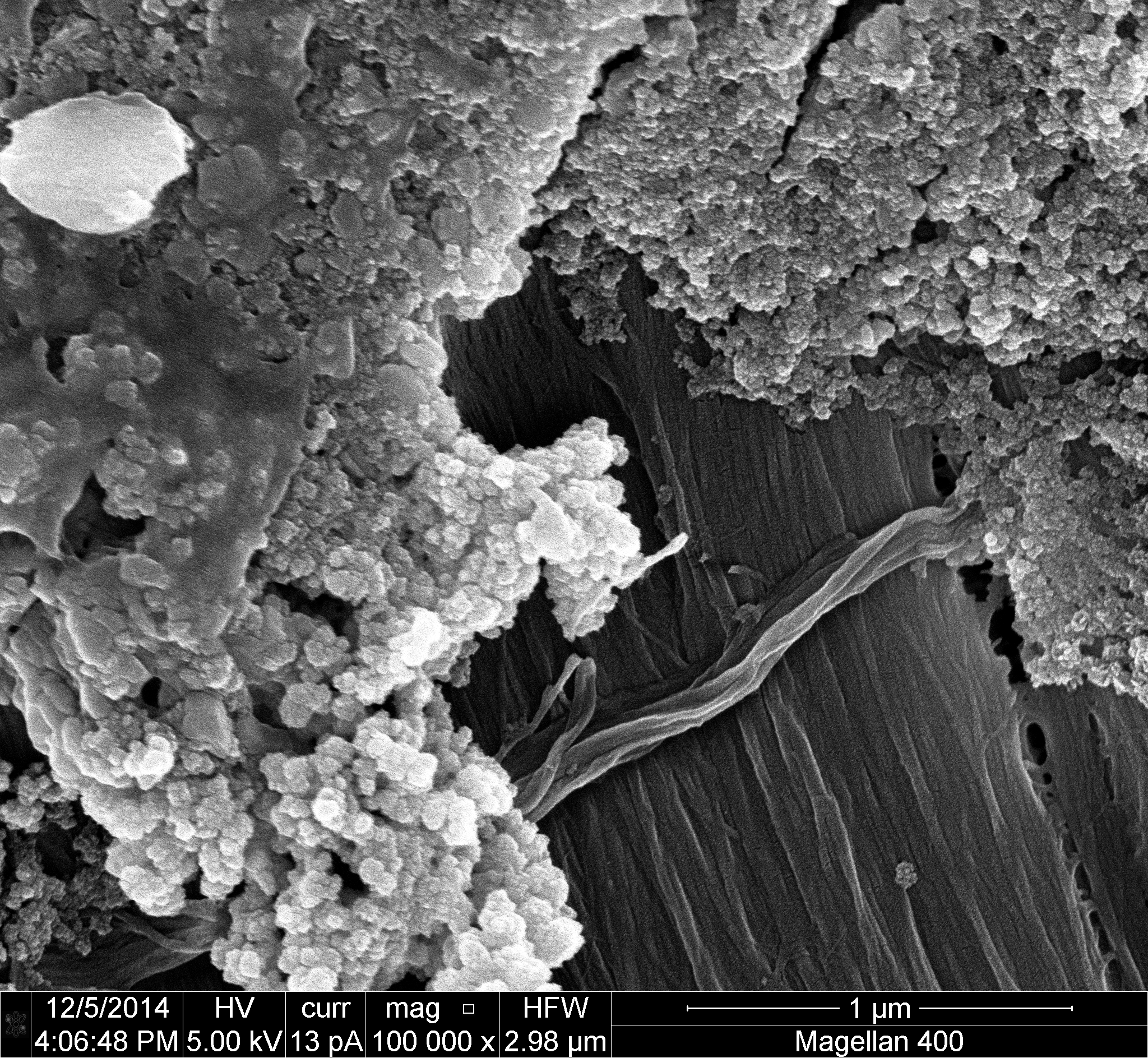  1 μm |
| --- | --- | --- |
| A. Paper | B. Paper with iron oxides | C. Paper with iron oxides after exposure to arsenic solution |

**S6 Fig. Iron oxides storage in paper fibers.** Scanning electron microscopy images. All images are to the same scale. A. An individual fiber of paper can be seen. B. When iron oxides are loaded onto the test card, they form clusters around the paper fibers. C. After adding the arsenic containing test solution, the iron oxides remain around the paper fibers.
