# Supplementary material for: Green design of a paper test card for urinary iodine analysis
Source: PLoS One. 2017 Jun 28;12(6):e0179716. doi: 10.1371/journal.pone.0179716 (PMC5489186; doi:10.1371/journal.pone.0179716)
Supplement: S7 Fig — (DOCX) [file pone.0179716.s010.docx]

**S7 Fig. Light box directions**

Find a completely sealed box with dimensions about equal to 12” x 8” x 5”. Line the inside of the box with white paper and secure with tape or glue. Secure 2 strands of LED lights to the inside of the box, either on the top or sidewalls. It is better if the lights are powered from an electrical outlet; battery-operated lights are highly discouraged as the light intensity will decrease over time as the battery discharges. Do not aim the lights directly at the imaging area as this will create a noticeable shadow. Cover the LED lights with as many layers of normal printing paper as necessary to diffuse the light (about 2-3 sheets should work). Cut a 1 cm diameter hole into the top of the box so that the camera lens can see into the box. Take a practice picture to check that no shadows are present. Adjust the position of the lights and the paper coverings as needed to get even dispersal of light.
